# Traumatic Diaphragmatic Hernia Treated With Minimally Invasive Abdominal and Thoracic Approach (MATA): A Case Report and Literature Review

**DOI:** 10.7759/cureus.78300

**Published:** 2025-01-31

**Authors:** Akira Toyoda, Osamu Murakami, Yoshio Ichihashi, Teruyoshi Amagai

**Affiliations:** 1 General Surgery, Yao Tokushukai General Hospital, Yao, JPN; 2 Respiratory Surgery, Yao Tokushukai General Hospital, Yao, JPN; 3 Clinical Engineering, Jikei University of Health Care Sciences, Osaka, JPN

**Keywords:** congenital bochdalek hernia, minimally invasive abdominal and thoracic approach (mata), minimally invasive laparoscopy, traumatic diaphragmatic hernia, video-assisted-thoracoscopy

## Abstract

We present a 43-year-old female who presented with left chest pain on exhalation after falling in her room five days ago. CT scan of the chest also revealed a suspected complicated diaphragmatic injury in the posterolateral hernia orifice measuring 34 × 27 mm with pneumothorax. Laparoscopy was performed in the lithotomy position with the left hand raised during thoracoscopy, followed by a thoracoscopic approach. Operative findings were as follows: 1) a left posterolateral diaphragmatic hernia (DH) was diagnosed, 2) the transverse colon was herniated into the thoracic cavity and appeared difficult to reduce laparoscopically due to visceral adhesion to the left lung, 3) thoracoscopy was added to release the fibrous adhesion of the colon to the lung, and 4) the diaphragmatic repair was performed with the running suture. In addition, we performed a literature review, which identified nine cases of DH treated with a minimally invasive abdominal and thoracic approach (MATA), including our case. From this experience, MATA is proposed as the first choice to treat DH when there is strong adhesion of herniated contents to the thoracic cavity viscera.

## Introduction

A diaphragmatic hernia (DH) is classified based on various factors, including congenital or acquired origin, trauma or iatrogenic causes, and whether it is symptomatic or asymptomatic [1]. Once the diagnosis of DH is made, surgery must be performed urgently, as delays may lead to complications such as volvulus, incarceration, and strangulation. The surgical approach can involve open surgery, laparoscopy, and/or thoracoscopy. In cases involving traumatic DH, where there is fibrous adhesion of herniated viscera to the lung, the thoracoscopic approach is preferred. However, this approach requires maintaining positive intra-thoracic pressure, which may compromise pulmonary and systemic circulation during the procedure. In urgent cases of traumatic DH with hemodynamic instability, it is questionable whether an assisted thoracoscopic procedure should be added. There are no randomized trials comparing the minimally invasive laparoscopic approach with or without thoracoscopic assistance [2]. In this case report, we present a traumatic DH successfully treated using minimally invasive abdominal and thoracic approach (MATA), which represents the ninth case treated by MATA reported in the literature.

## Case presentation

A 43-year-old female presented with left chest pain on breathing, which had developed after she fell in her room 5 days ago. Immediately after the fall, the patient visited a local doctor, complaining of dyspnea and decreased SpO2, and was prescribed an analgesic. However, the left chest pain on respiration persisted, and the patient was transferred to our hospital. She had mental retardation.

**Figure 1:**
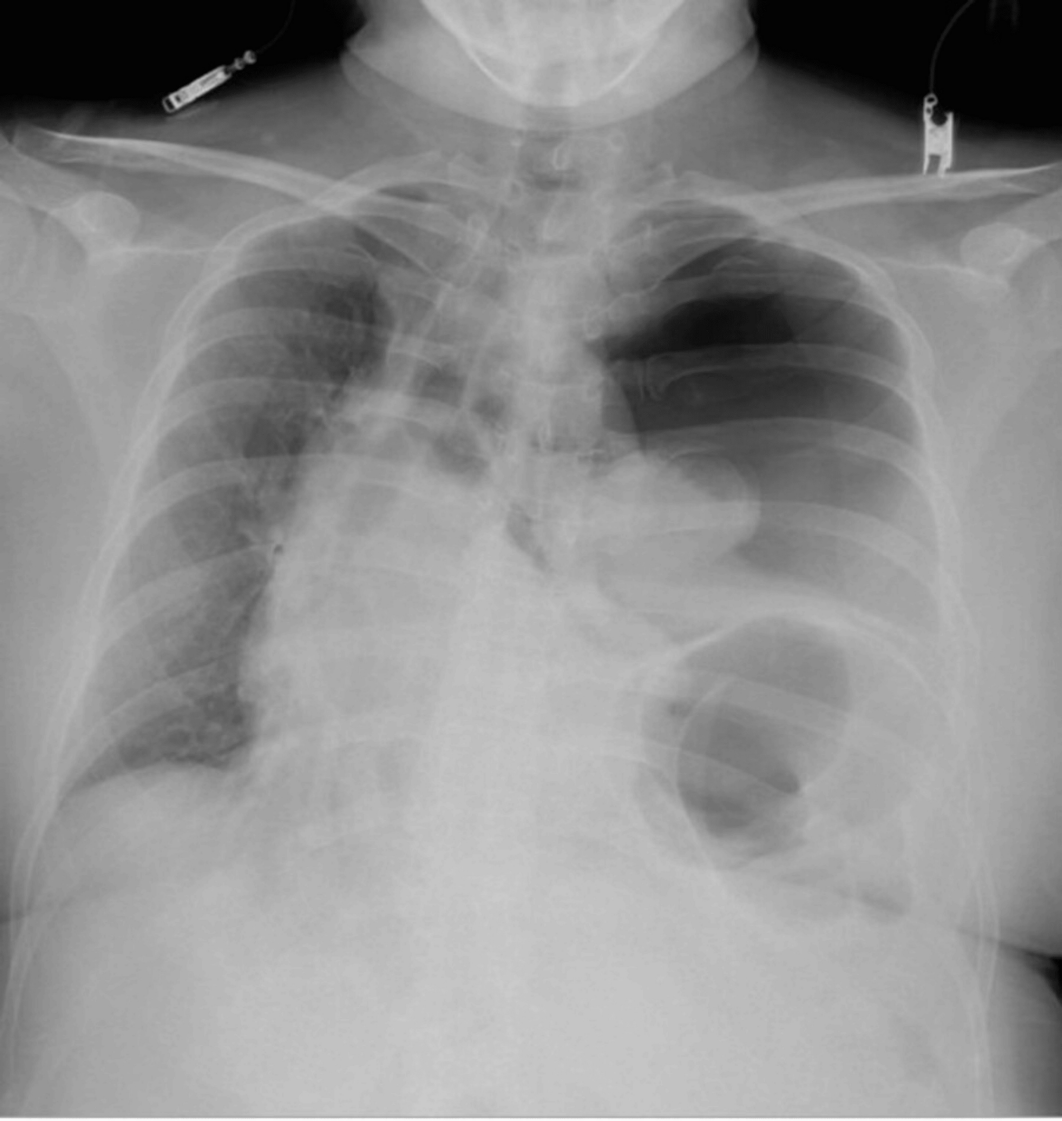
Chest X-ray This radiograph shows a shift of the heart and mediastinum to the right. Based on these findings, a left pneumothorax was diagnosed.

**Figure 2:**
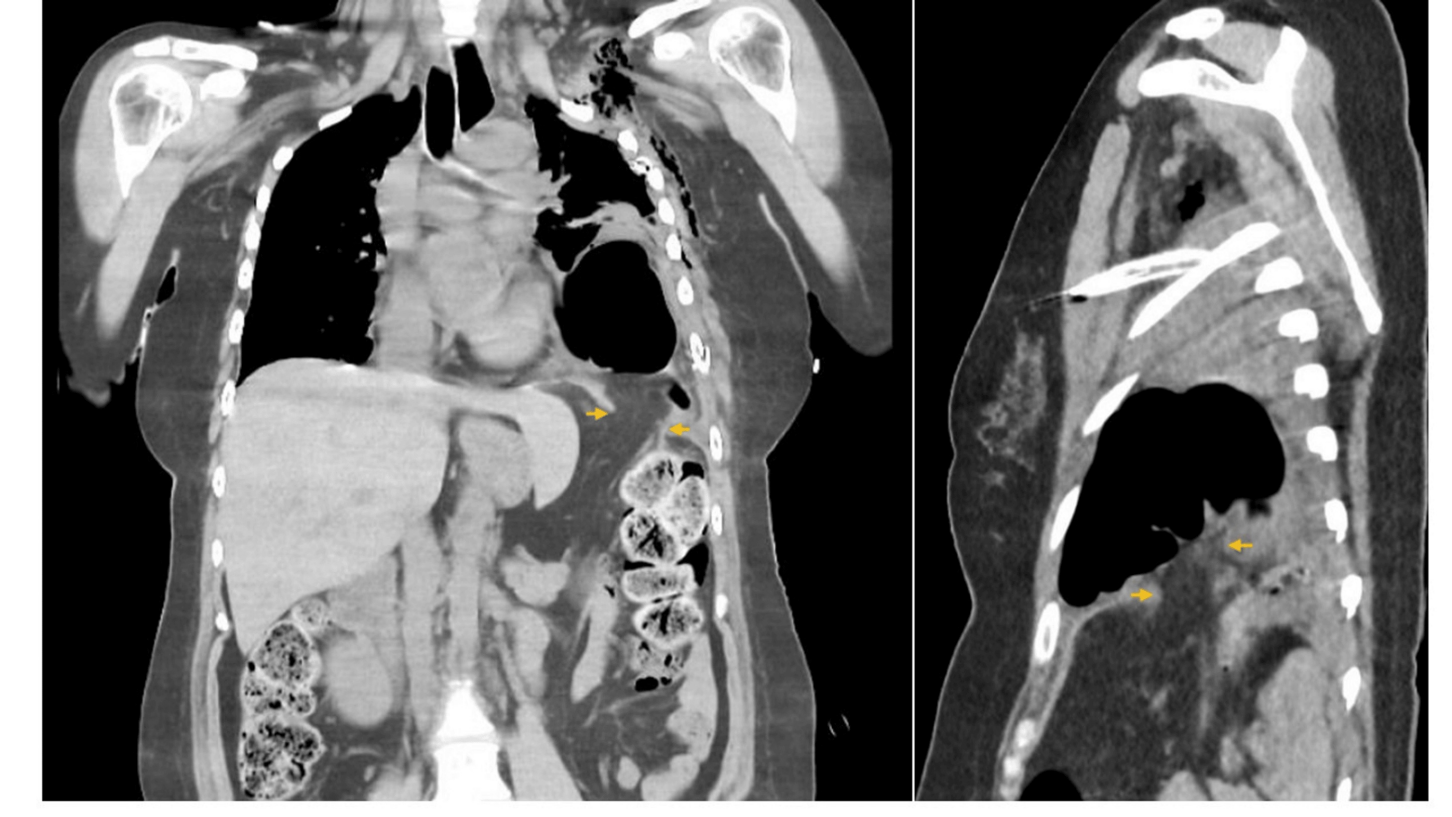
CT images of the chest Left: Frontal view; Right: Sagittal view. The arrows indicate the posterolateral hernia orifice and the herniated colon in the thoracic cavity.

On physical examination, breath sounds were absent over the left chest. Her vital signs were as follows: body temperature 36.4°C, blood pressure 105/70 mmHg, heart rate 105 beats per minute, and SpO2 93%. Blood tests revealed leukocytosis, anemia, hypoalbuminemia, and elevated C-reactive protein (Table 1). A chest X-ray showed left pneumothorax (Figure 1). A chest CT scan revealed gastrointestinal gas in the left lower lung field and a posterolateral diaphragmatic defect measuring 34 x 27 mm, with suspected pneumothorax (Figure 2).

**Table 1:** The results of ABG analysis, CBC, and serum chemistry on the first visit Alb: albumin, ALT: alanine transferase, AST: asparagine transferase, BS: blood sugar, BUN: blood urea nitrogen, CRP: C-reactive protein, HbA1c: hemoglobin A1c, Hb: hemoglobin, HCO_3_^-^: bicarbonate, Ht: hematocrit, PaCO_2_: arterial partial pressure of carbon dioxide, PaO_2_: arterial partial pressure of oxygen, Plt: platelet, RBC: red blood cell, T-bil: total bilirubin, WBC: white blood cell, ABG: arterial blood gas, CBC: complete blood count

	Result	Reference
pH	7.406	7.36～7.46
PaCO_2_	36.6	34～46 mmHg
PaO_2_	69.7	80～110 mmHg
HCO_3_^-^	22.5	23～31 mmol/L
WBC	18,800	3,300～8,600 count/μL
RBC	349	386～492 × 104/μL
Hb	8.5	11.6～14.8 g/dL
Ht	28.3	35.1～44.1%
Plt	32.4	15.8～34.8 × 10^4^/μL
CRP	17.27	～0.14 mg/dL
Alb	2.7	4.1～5.1 g/dL
T-bil	0.5	0.4～1.5 mg/dL
AST	16	13～30 U/L
ALT	11	7～23 U/L
BS	132	73～100 mg/dL
BUN	8.1	8.0～20.0 mg/dL
Creatinine	0.67	0.46～0.79 mg/dL
Na	126	138～145 mEq/L
K	3.4	2.6～5.5 mEq/L
Cl	93	101～108 mEq/L

Based on her history, physical examination, and X-ray findings, a traumatic left DH with pneumothorax was diagnosed. A chest tube was placed into her left thoracic cavity, draining 900 mL of blood. A laparoscopic repair was scheduled. Preoperatively, we discussed that if the laparoscopic repair proved difficult or inadequate, a thoracoscopic approach would be considered. The lithotomy position was chosen, with the left arm raised for thoracoscopy in the reverse Trendelenburg position. To prevent left lung injury, anesthesia was managed with right-sided single-lung intubation.

**Figure 3:**
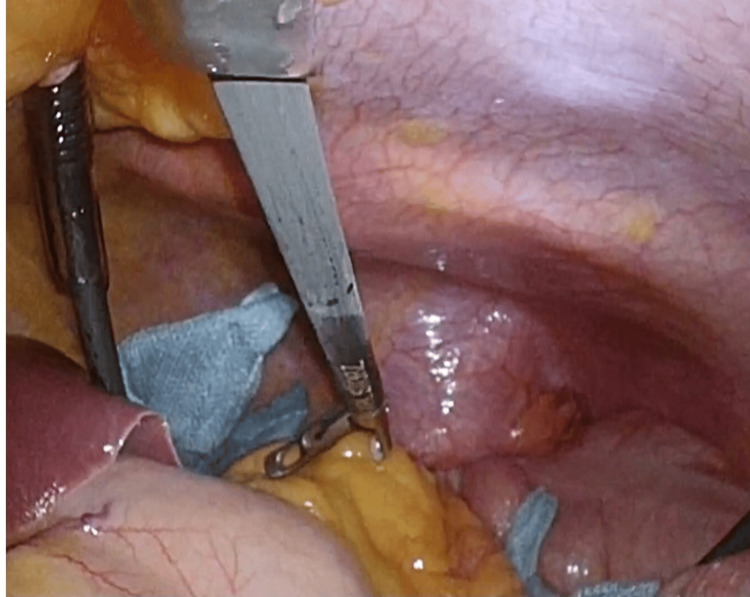
Intraoperative findings from the laparotomy view At laparotomy, the findings were as follows: 1) the edge of the diaphragmatic hernia (DH) appeared scarred, 2) reduction of the transverse colon through the DH orifice could not be completed, 3) a thoracoscopic approach was added, and reduction of the herniated transverse colon was completed after repairing the perforated colon. The image above shows the mesocolon herniated through the DH orifice. DH, diaphragmatic hernia

A laparoscopy was performed, and a left posterolateral diaphragmatic hernia (DH) was diagnosed (Figure 3). The hernia contents, which included the transverse colon, appeared difficult to reduce laparoscopically due to visceral adhesion to the left lung. Therefore, a thoracoscopy, performed simultaneously with laparoscopy (referred to as MATA), was added. Direct diaphragmatic repair with running sutures using absorbable stitches was successfully performed after pleuroperitoneal lavage. A chest tube and peritoneal drain were placed. The patient was weaned from mechanical ventilation, and both the chest tube and peritoneal drain were removed on the 4th postoperative day (POD). The patient was discharged home uneventfully on the 11th POD.

## Discussion

MATA as the surgical approach for adhesive DH

In general, the laparoscopic approach is preferred for DH repair. The advantages of adding a thoracic approach include the creation of pneumothorax, which allows for better visualization and facilitates the reduction of herniated contents. Diaphragmatic defect closure and bleeding control have been reported as easier and safer with combined laparoscopic and thoracoscopic approaches [3,4], with low-pressure inflation-assisted bowel reduction achieved [5,6]. The pressure gradient created by the combined MATA approach could be an advantage worth recognizing.

Literature review of cases with DH treated using MATA

Based on a comprehensive search of the literature review, nine cases, including ours, treated by MATA were collected (Table 2) [7-13]. To the best of our knowledge, this literature review is the first overview of MATA for DH treatment. Among the nine cases of DH, four were due to trauma and three were congenital. All except for ours were male, with ages ranging from 17 to 78 years. Despite the varying causes and ages of the DH cases, we would like to emphasize MATA as a surgical option for DH, particularly when there is massive adhesion of the herniated tissue to the thoracic organs.

**Table 2:** The results of the literature review of patients with DH treated by MATA MATA, minimally invasive abdominal and thoracic approach; DH, diaphragmatic hernia

Case	Age	Sex	Side	Cause	Traumatic	Herniated organs	Size of hernia orifice (mm)	Year	References
1	17	Male	Left	Congenital Bochdalek	-	Left colon, spleen	50 × 60	2010	Tokumoto et al. [7]
2	45	Male	Left	Unknown	-	Small intestine, spleen, colon	100 × 80	2020	Chauhan et al. [8]
3	29	Male	Left	Diaphragmatic eventration	-	Stomach, colon	120 × 100	2020	Chauhan et al. [8]
4	26	Male	Left	Congenital Bochdalek	-	Omentum	40 × 30	2021	Nambara et al. [9]
5	47	Male	Left	Chest trauma	+	Omentum	40 × 20	2023	Yoshimine et al. [10]
6	78	Male	Right	Rupture of the diaphragm eventration	-	Right lobe of the liver, gall bladder, right colon, duodenum, stomach, omentum	30 × 50	2024	Gupta et al. [11]
7	34	Male	Left	Motor vehicle accident/intrathoracic kidney	+	Left kidney	7.5 × 2.5	2024	Shenoy et al. [12]
8	45	Male	Right	Congenital Bochdalek	-	Left lobe of the liver		2024	Mikami et al. [13]
9	43	Female	Left	Fell down, mental retardation	+	Transverse colon	34 × 27	2025	Toyoda et al.

## Conclusions

A 43-year-old female with a blunt traumatic left-sided DH underwent repair using MATA. From the perspective of utilizing the pressure gradient with the combined laparoscopic and thoracoscopic procedure, it appears that MATA is an acceptable first choice for surgical repair of DH when there are adhesions of the hernia contents within the thoracic cavity. Our case is the ninth case of CD treated with MATA in the literature.
